# Uncovering the impact of the cardiovascular system on cerebrovascular health using MRI

**DOI:** 10.1113/EP092467

**Published:** 2025-09-03

**Authors:** Ian D. Driver, Kevin Murphy

**Affiliations:** ^1^ Cardiff University Brain Research Imaging Centre (CUBRIC), School of Physics and Astronomy Cardiff University Cardiff UK

**Keywords:** arterial stiffness, cerebral blood flow, vascular tone

## Abstract

Human cerebrovasculature is finely tuned to enable local changes in blood flow to meet the brain's demands, whilst protecting the brain from systemic changes in blood pressure, both acutely during a heartbeat and chronically over time. This review summarises cerebrovascular structure and function, their role in disease and neurodegeneration and the part MRI measurements can play in probing them. MRI methods to measure various aspects of cerebrovascular physiology are described and placed in context of applications studying cerebrovascular health. The role of the cardiovascular system linking the cardiac pulse wave to cerebrovascular disease and gaps in mechanistic knowledge are highlighted.

## Abstract (highlights)

- **What is the topic of this review?**
  This review considers MRI measurements of the human cerebrovasculature and how they are impacted by the cardiovascular system.
- **What advances does it highlight?**
  This review article appraises the current state‐of‐the‐art MRI measurements of cerebrovascular function in the context of healthy ageing and disease and links them to underlying cardiovascular factors.

## 1 CEREBROVASCULAR FUNCTION

The human cerebrovasculature supplies the metabolic substrates required to meet the brain's energetic demands (Bordone et al., 2019; Camandola & Mattson, 2017), whilst being able to react to local increases in demand through functional hyperaemia, that is, corresponding local increases in blood flow (Buxton, 2021; Fox & Raichle, 1986). Recently, the cerebrovascular network has come to be thought of as playing a key role in the brain's waste clearance system (Agarwal & Carare, 2021; Iliff et al., 2012), with a mechanism postulated whereby transport occurs through perivascular spaces, driven by vasomotion and pulsatility of blood vessels.

**FIGURE 1:**
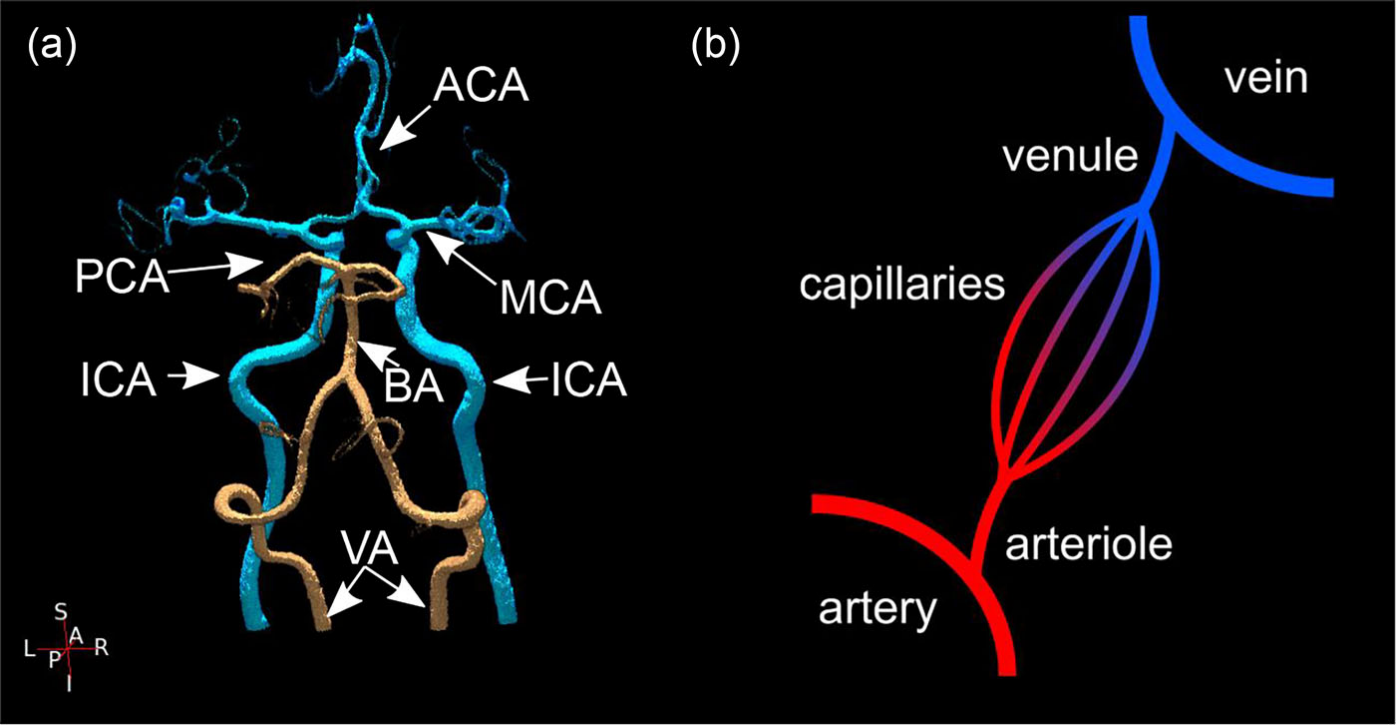
(a) An example of the predominant configuration of the circle of Willis, with the posterior circulating arteries in copper and the anterior circulating arteries in light blue. ACA, anterior cerebral artery; BA, basilar artery; ICA, internal carotid artery; MCA, middle cerebral artery; PCA, posterior cerebral artery; VA, vertebral artery. (b) Illustration of the cerebrovascular tree. MRI measures of the microvasculature (arterioles, capillaries and venules) are covered in Sections 3 and 4. MRI measures of the macrovasculature (feeding arteries and draining veins) are covered in Section 5.

The anatomy of the cerebrovasculature is covered extensively elsewhere (Cipolla MJ., 2009; Duvernoy et al., 1981), but a brief summary is given here. The brain's blood supply is fed by the left and right internal carotid and vertebral arteries. The vertebral arteries converge into the basilar artery, supplying the brainstem and cerebellum. The internal carotid arteries and basilar artery feed the circle of Willis, which supplies the cerebral cortex via the anterior, middle and posterior cerebral arteries (Figure 1a). These arteries branch into smaller arteries, then arterioles, which supply the capillary bed, from which most of the exchange of metabolic substrates occurs with perfused tissue. An MRI technique called arterial spin labelling (ASL) can be used to track blood from the larger cerebral arteries as it perfuses into tissue (Okell et al., 2019; van Harten et al., 2021; Wong & Guo, 2012). The circle of Willis allows collateral flow, so in the case of abnormal arterial anatomy or occluded vessels, the regions perfused by each artery can adapt to preserve local blood flow (Helle et al., 2013; Wu et al., 2008). The capillary bed is drained by venules, which feed into a series of larger veins, until they drain into the venous sinuses. Recently, MRI has been used to form atlases of arterial (Bernier et al., 2018; Mouches & Forkert, 2019) and venous (Bernier et al., 2018; Huck et al., 2019) structures across healthy brains, suggesting more spatial variability in venous than arterial anatomy.

Cerebral blood vessels are lined with endothelial cells, which form the blood–brain barrier, regulating the exchange of metabolic substrates and waste products. The blood–brain barrier prevents macromolecules in blood plasma from entering the brain, whilst maintaining the chemical environment required for healthy metabolism (Abbott et al., 2010; Zlokovic, 2008).

A key component of cerebrovascular function is the concept of neurovascular coupling. Arterial vascular tone is controlled by smooth muscle cells, which constrict and relax to change vascular resistance through vessel diameter changes and, ultimately, blood flow. Smooth muscle cells react to neuronal signalling, leading to a localised vasodilatation in the arteries and arterioles feeding the site of neuronal activation (Iadecola, 2017; Stackhouse & Mishra, 2021). This vasodilatation increases perfusion to the capillary bed, providing an increase in metabolic substrates, including oxygen transported by oxygenated haemoglobin. The increased oxygen delivery exceeds the increased oxygen extraction arising from an elevated oxygen metabolism (Ito et al., 2005), so the oxygenation of veins draining the site of neuronal activation is, counterintuitively, increased (Fox & Raichle, 1986). Increased venous oxygenation means less deoxygenated haemoglobin, which provides the source of contrast (Ogawa et al., 1990) for blood oxygenation‐level‐dependent functional MRI (BOLD fMRI). As such, BOLD fMRI demonstrated local signal increases in response to task performance (Bandettini et al., 1992; Kwong et al., 1992; Ogawa et al., 1992), which has since been extensively used as a surrogate measurement for mapping neuronal responses.

The cerebrovasculature cannot be treated in isolation from the rest of the cardiovascular system, with the aorta feeding the internal carotid and vertebral arteries (via the common carotid and subclavian arteries, respectively). Therefore, aortic stiffness will directly affect blood flow (Jefferson et al., 2018) and energy of the cardiac pulse wave entering the cerebrovasculature (Tarumi et al., 2014). The aorta stiffens throughout the adult lifetime, especially beyond middle‐age (Parikh et al., 2016). Aortic stiffness is associated with hypertension and diabetes (Cavalcante et al., 2011; Cruickshank et al., 2002), and aortic stiffness may cause hypertension (Najjar et al., 2008). Aortic stiffness is lower in athletes, compared to those with a sedentary lifestyle, and aortic stiffening appears to be reduced by aerobic exercise (Ashor et al., 2014; Lavie et al., 2015; Li et al., 2023; Seals, 2014). Considering aerobic exercise training can also improve cognitive performance in participants with mild cognitive impairment, concurrently with reductions in carotid arterial stiffness and cerebrovascular reactivity (Penukonda et al., 2025), furthering understanding of the cerebrovasculature will help to link cardiovascular health to the healthy function and decline of the ageing brain. The interaction of aortic and cerebrovascular function is further explored in Section 5.3.

In this review we discuss how MRI can measure various aspects of the cerebrovasculature. Section 2 provides examples where cerebrovascular dysfunction has a role in the pathophysiology of a range of diseases. Section 3 provides an overview of MRI measures for studying the brain's microvasculature (capillaries, arterioles and venules, see Figure 1b). Section 4 provides examples of applications of these microvascular MRI measures, which are furthering understanding of the role of the cerebrovasculature in sustaining a healthy brain. Section 5 summarises MRI measures of the macrovasculature (arteries and veins, see Figure 1b) and the role the cardiovascular system can have on cerebrovascular function, highlighting the role of cardiovascular health in neurodegeneration and the gaps in knowledge required to develop successful interventions to prevent or reverse this neurodegeneration.

## 2 CEREBROVASCULAR HEALTH

Cerebrovasculature has a role in most diseases that affect the brain, although it is often unclear whether cerebrovascular mechanisms are causative or consequential. In this section, we briefly focus on disorders where the cerebrovasculature has a clear and major contribution to disease aetiology.

In 2021, there were over 100 million incidences of stroke globally, with approximately two‐thirds being ischaemic and one‐third being haemorrhagic (Feigin et al., 2024). Whilst the main risk factors overlap with cardiovascular risk factors (Tsao et al., 2023; Vangen‐Lønne et al., 2017), a wide range of cerebrovascular diseases have also been identified as risk factors for stroke, such as atherosclerosis, aneurysm and arteriovenous malformation (Juttukonda & Donahue, 2019). Small vessel disease is a group of pathologies affecting the perforating arteries and arterioles in the brain (Wardlaw et al., 2013). Broadly, small vessel disease is either local occlusion or haemorrhage, leading to white matter hyperintensity or lacune, which are detectable using MRI. Generally, cerebrovascular diseases lead to neurodegeneration through infarction (tissue death due to impaired blood supply) and breakdown of the blood–brain barrier. White matter hyperintensities, also known as leukaraiosis, present as regions of low signal in computed tomography (CT) and high signal in some MRI contrasts (Wardlaw et al., 2015). They are generally considered to be a form of small vessel disease (Wardlaw et al., 2013), often found with lacunes and containing blood‐born proteins that do not typically cross the blood–brain barrier (Wardlaw et al., 2015). White matter hyperintensities are associated with cognitive decline, risk of stroke, dementia and, ultimately, mortality (Debette & Markus, 2010).

In hypertension, vascular resistance is increased to preserve cerebral blood flow (CBF) in the presence of high systemic blood pressure (Kety & Hafkenschiel, 1948; Warnert, Rodrigues et al., 2016). The higher vascular resistance leads to less compliance, that is, less capability to dilate to meet spontaneous increases in demand. This can lead to neurodegeneration and cognitive decline, whilst being a major risk factor for both dementia and stroke (Faraco & Iadecola, 2013). There is controversy as to the aetiology of hypertension, as to whether there is a cerebrovascular origin, with hypoperfusion to the autonomic centres in the brainstem leading to increased sympathetic nerve activation, increasing blood pressure (Grassi et al., 2015; Hart, 2016; Warnert, Rodrigues et al., 2016). Hypoperfusion has been suggested to arise from abnormal arterial anatomy in the vertebral arteries, such as vertebral artery hypoplasia, or variants to the circle of Willis (Warnert, Rodrigues et al., 2016).

Breakdown of the blood–brain barrier is implicated in several diseases. The blood–brain barrier prevents harmful chemicals from entering the central nervous system (CNS), whilst having a role in removing metabolic waste products (Abbott et al., 2010; Zlokovic, 2008). Blood–brain barrier dysfunction is linked to build‐up of amyloid‐beta in Alzheimer's disease (Deane et al., 2003; Sweeney et al., 2018), with an association between amyloid‐beta and prevalence of micro‐bleeds (Kantarci et al., 2013). In multiple sclerosis, immune T‐cells cross the blood–brain barrier, leading to an autoimmune response and inflammation (Correale & Villa, 2007). Inhibited clearance of waste products has been observed in Parkinson's disease (Bartels et al., 2008; Kortekaas et al., 2005), whilst blood‐borne proteins have been found in *ex vivo* CNS tissue in Alzheimer's disease, Parkinson's disease, Huntington's disease, amyotrophic lateral sclerosis and multiple sclerosis (Sweeney et al., 2018).

## 3 MEASURING MICROVASCULAR FUNCTION WITH MRI

MRI is a very versatile modality for imaging different aspects of the cerebrovasculature, exploiting the magnetic properties of blood and tissue to provide different sources of signal contrast depending on the physiological parameter of interest. This section highlights some of the methods used for studying the brain's microvasculature (arterioles, capillaries and venules) and how they interact with the tissue that they perfuse. Table 1 summarises each MRI method and presents their strengths and weaknesses in context of alternative methods.

**TABLE 1:** Summarising the key strengths and limitations of each MRI method in context of alternative techniques.

Cerebrovascular parameter	MRI modality (text section covered)	Vessels studied	Strengths and weaknesses
Cerebral blood flow	ASL (3.1)	Capillaries/tissue	+ Avoids exogenous contrast agents, such as radiotracers (PET/CT) or gadolinium chelates (contrast‐enhanced MRI) + Flexibility to resolve dynamic changes in perfusion − Lower sensitivity than PET or contrast‐enhanced MRI − Challenging to measure in white matter, due to lower perfusion and longer arrival times than grey matter
	pcMRI (5.1)	Arteries	+ Spatial sensitivity to resolve arteries within the skull + Not affected by wave refraction through the skull, as affects TCD − Lower temporal resolution than TCD
Oxygen metabolism	Calibrated fMRI (3.3)	Capillaries/tissue	+ Avoids use of radioactive tracers, used in PET + Finer spatial resolution than PET − Requires inhalation of CO_2_ and O_2_ through a close‐fitting facemask or mouthpiece, which can be uncomfortable
	Tissue susceptibility and relaxometry (3.3)	Capillaries/tissue	+ Avoids use of radioactive tracers, as used in PET + Finer spatial resolution than PET − May be biased by local non‐blood susceptibility sources and non‐local magnetic field inhomogeneities
	Vein susceptibility and relaxometry (5.2)	Veins (e.g. superior sagittal sinus)	+ Spatial sensitivity to resolve veins within the skull + Not affected by wave reflection by the skull, as affects fNIRS − Lower temporal resolution than fNIRS
Blood–brain barrier (BBB)	Water exchange (ASL or diffusion; 3.5)	Capillaries/tissue	+ BBB breakdown detectable at an earlier stage with water permeability than macromolecules + Avoids exogenous contrast agents, such as radiotracers (PET/CT) or gadolinium chelates (contrast‐enhanced MRI) − Lower sensitivity than PET or contrast‐enhanced MRI
Arterial stiffness (PWV)	4D flow pcMRI (5.3)	Arteries (CoW)	+ Spatial sensitivity to resolve arteries within the skull + Not affected by wave refraction through the skull, as affects TCD + Resolves pulse waveforms across the whole CoW concurrently − Takes several minutes to acquire an image, so averages over several hundred heartbeats
	Inflow MRI (5.3)	Arteries (CoW)	+ Spatial sensitivity to resolve arteries within the skull + Not affected by wave refraction through the skull, as affects TCD + Able to resolve the pulse waveform for individual heartbeats − Method limited to a few slices, so cannot measure across the whole CoW concurrently

Each method is discussed in detail in the respective main text section. ASL, arterial spin labelling; BBB, blood–brain barrier; CoW, circle of Willis; CT, computed tomography; fNIRS, functional near‐infrared spectroscopy; pcMRI, phase contrast MRI; PET, positron emission tomography; PWV, pulse wave velocity; TCD, transcranial Doppler ultrasound.

### 3.1 Cerebral blood flow

**FIGURE 2:**
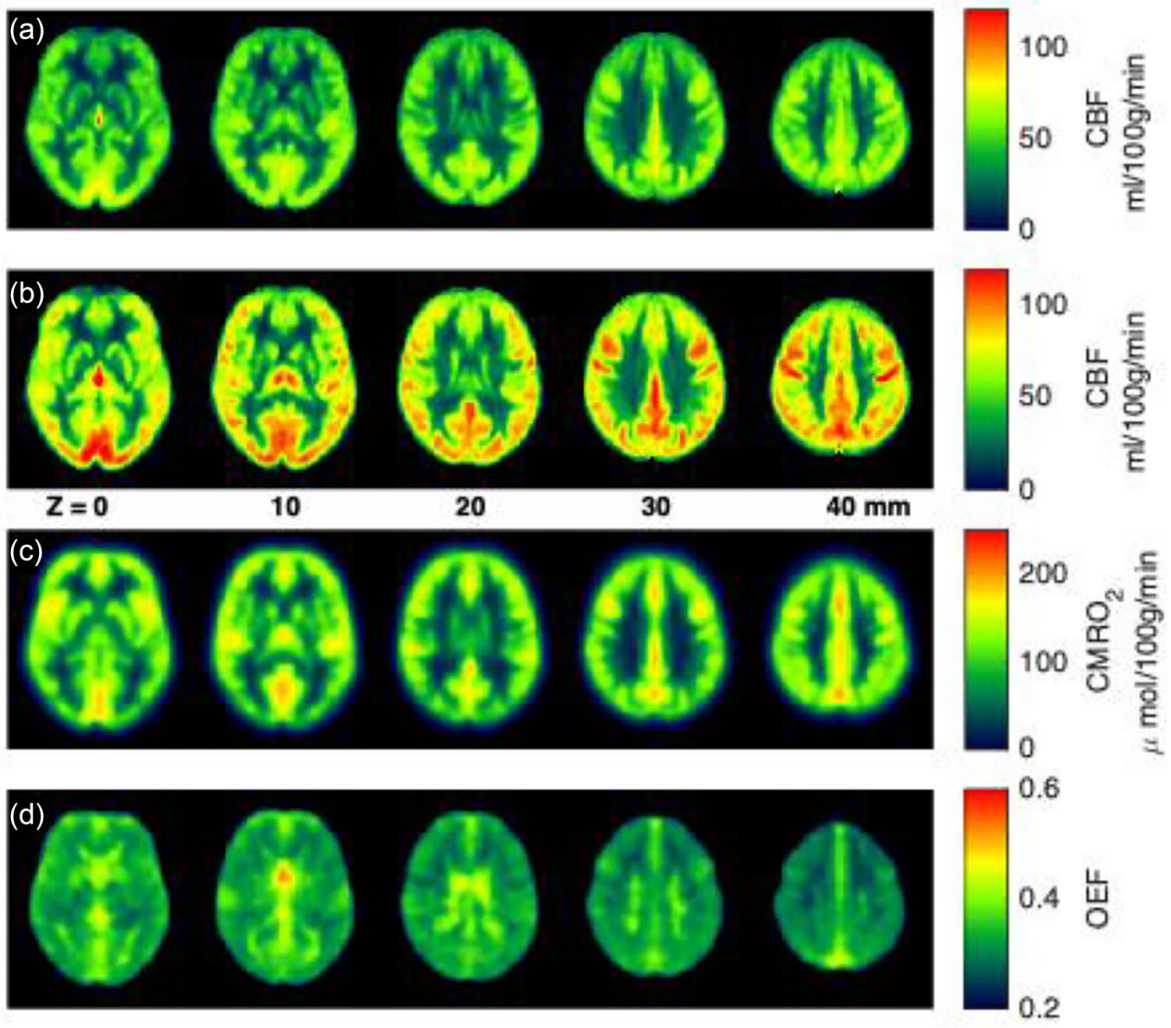
Example images for microvascular mapping MRI methods. (a) Example CBF map acquired with ASL. (b) The respective CBF map where participants inhaled 5% CO_2_, inducing vasodilatation. The change in CBF from (a) to (b) is used to calculate CVR (when normalised by the change in arterial CO_2_). Data and methods for (a) and (b) were presented previously (Driver et al., 2025). (c, d) Example CMRO_2_ map (d) and example OEF map (d), based on the breath hold calibrated fMRI method, with data and methods presented previously (Driver et al., 2024). ASL, arterial spin labelling; CBF, cerebral blood flow; CMRO_2_, cerebral metabolic rate of O_2_; CVR, cerebrovascular reactivity; OEF, oxygen extraction fraction (the fraction of oxygenated haemoglobin converted to deoxygenated haemoglobin in the capillary bed).

In the context of MRI research, CBF refers to perfusion and is defined as the volume of blood that perfuses a unit mass of tissue per unit time, with common units of mL/100 g/min (50–70 mL/100 g/min are typical values for healthy grey matter). Whilst CBF can be mapped using radioactive tracers using positron emission tomography (PET) (Fan et al., 2015) or CT (Takemaru et al., 2017), or with an exogenous MRI contrast agent (Østergaard et al., 1998), MRI also provides an endogenous means to non‐invasively map CBF, called arterial spin labelling (ASL). In ASL, water in arterial blood is labelled, then, after a time for the blood water to perfuse into tissue, an image is acquired, whereby the labelled water provides perfusion contrast. Historically, several methods and locations for the labelling of arterial blood water have been developed in parallel (Dai et al., 2008; Detre et al., 1992; Edelman et al., 1994; Kim, 1995; Williams et al., 1992; Wong et al., 1998, 2006). With a view to promoting clinical adoption, the ASL research community have come together with consensus papers which provide focussed recommendations for clinical use (Alsop et al., 2015; Qin et al., 2022). The recommendations are to use a labelling technique called pseudo‐continuous ASL (pCASL), whereby arterial blood water is labelled as it passes through a labelling plane. The labelling plane is positioned at the level of the internal carotid and vertebral arteries, at a position where both pairs of arteries are approximately straight and parallel, such that a labelling plane can be positioned perpendicular to all four arteries. However, in the case of long arterial arrival times, such as in some cerebrovascular diseases (Bokkers et al., 2009; Bolar et al., 2019; Fan et al., 2017; Qiu et al., 2012; D. J. J. Wang et al., 2013) and healthy ageing (Mahroo et al., 2024; Scheel et al., 2000), this approach can be biased by the late arrival of the labelled blood. In these cases, an alternative ASL approach is more appropriate, whereby the labelling is applied by velocity‐encoding, which can label arterial blood water much closer to the site of perfusion than the spatially selective pCASL (Qin et al., 2022; Wong et al., 2006). Figure 2a demonstrates example CBF maps from ASL data.

### 3.2 Cerebrovascular reactivity

A key advantage of ASL over exogenous tracer‐based measurements of CBF is that changes in CBF can be observed over the time scale of several seconds, whereas tracer‐based measurements are limited by the kinetics of the delivery of the tracer. Measuring changes in perfusion on a finer time scale allows measurement of cerebrovascular reactivity (CVR), which is a key measure of the capacity of the cerebrovasculature to react to local changes in demand. CBF CVR can be measured directly using ASL, or indirectly, using BOLD fMRI (Smeeing et al., 2016). CBF CVR has lower sensitivity but provides a direct measure of vascular reactivity. BOLD CVR has greater sensitivity, but has less specificity, measuring the combination of oxygenation and blood volume changes of veins draining the tissue of interest. Various vasoactive challenges can be used to measure CVR, with a detailed overview provided previously (Fierstra et al., 2013; Pillai & Mikulis, 2015). Acetazolamide is administered intravenously and is a potent cerebral vasodilator (Vagal et al., 2009; Vorstrup et al., 1986; Zhao et al., 2021), but the vasodilatory effect is not easily reversable. Arterial CO_2_ concentration also acts as a potent vasodilator (Reivich, 1964) and can be modulated either by inhalation of air supplemented by a small percentage of CO_2_ (Blockley et al., 2011; Kastrup et al., 2001; Slessarev et al., 2007; Wise et al., 2007), or by modifying breathing frequency and/or depth (Bright & Murphy, 2013; Bright et al., 2009; Kastrup et al., 1998). The advantage of modulating arterial CO_2_ is that the effect can be easily reversed on the order of seconds, by returning to normal breathing conditions, allowing repeated block paradigms and minimising risk of adverse physiological reactions to the hypercapnia (Gitelman et al., 1991; Woods et al., 1988). The simplest method for modulating arterial CO_2_ is for the participant to hold their breath for a short period (15–30 s), or change breathing pace or depth. This is achieved in the MRI environment by providing visual or auditory cues at the desired timings. These methods are simple to implement, not requiring dedicated hardware, and are generally well tolerated by participants. However, these respiratory challenges give transient MRI signal changes and there is a large variability in task performance affecting the individual changes in arterial CO_2_. Monitoring exhaled CO_2_ levels can somewhat mitigate for task performance variability (Bright & Murphy, 2013). Modulating inhaled CO_2_ can be used to provide steady‐state changes, which are more conducive to a robust estimation of MRI signal changes required to calculate CVR. These are contrasted to air, or a gas mixture matching air (21% O_2_, 79% N_2_, without CO_2_). However, the inhaled gas mixtures are delivered through close‐fitting face masks or mouthpieces that can cause some participant discomfort, whilst these methods require dedicated hardware and expertise that are not always available in either research or clinical settings. Broadly, inhaled gas methods can be divided into fixed‐inspired (Kastrup et al., 1998) and computer‐controlled targeting methods (Slessarev et al., 2007; Wise et al., 2007). Fixed‐inspired gas delivery involves a set percentage of CO_2_ delivered to the participant, commonly 5%. This approach does not account for individual physiological differences (ventilation and metabolism), so induces different changes in arterial CO_2_ across participants. Computer‐controlled targeting of CO_2_ involves dynamically changing inhaled gas mixtures based on either feedback of exhaled CO_2_, or on a prospective model based on the participant's initial physiological state. These methods can produce more repeatable changes in arterial CO_2_ than fixed‐inspired methods, but require careful and time‐consuming initial calibration measurements, which require operator experience to avoid drifts in CO_2_ levels across the experiment. Figure 2a shows an example CBF map when breathing air and Figure 2b shows the corresponding CBF map when participants breathed in a gas mixture with 5% CO_2_, causing vasodilatation. The resulting CBF increase is used to calculate CVR, by dividing the change in CBF by the change in arterial CO_2_.

### 3.3 Oxygen metabolism

Local oxygen metabolism can be mapped with MRI. One approach, termed dual‐gas calibrated fMRI, measures the CBF, and oxygenation responses to hypercapnia (inspired CO_2_) and hyperoxia (increasing inspired O_2_) to map oxygen extraction from the tissue capillary bed (Bulte et al., 2012; Gauthier et al., 2012; Wise et al., 2013). Recently, this approach has been extended by considering oxygen diffusion between capillaries and mitochondria (Germuska et al., 2019), which allows oxygen extraction to be mapped using only hypercapnia (Chiarelli et al., 2022) and even from breath holding (Driver et al., 2024), which avoids the need for a complicated set‐up for delivery of exogenous gases and associated participant discomfort. Example maps of oxygen metabolism and extraction fraction are shown in Figure 2c and d. From a similar motivation of avoiding complicated experimental set‐up and participant discomfort associated with delivering exogenous gas challenges, other MRI approaches have been developed to measure basal cerebral oxygen extraction, without the need for a perturbation. These methods make use of the effect of deoxygenated haemoglobin on the local magnetic environment, with deoxygenated haemoglobin having a higher magnetic susceptibility than both oxygenated haemoglobin and the surrounding tissue environment (Spees et al., 2001). Oxygen extraction is mapped either by measuring local susceptibility using a technique called quantitative susceptibility mapping (QSM), or by characterising the transverse relaxation of the MRI signal, or by a combination of both contrasts (An & Lin, 2000; Cherukara et al., 2019; Cho et al., 2021; He & Yablonskiy, 2007; Küppers et al., 2022; Lee & Wehrli, 2022; Ulrich & Yablonskiy, 2016). Challenges of these methods include how to distinguish blood oxygenation from blood volume, their inability to resolve susceptibility contributions from other sources, such as non‐haem iron and myelin, and sensitivity to bias from non‐local contributions to the magnetic field (Christen et al., 2014). However, once these issues are overcome, the advantage of not relying on exogenous gas challenges makes these approaches more promising for clinical translation.

### 3.4 Cerebral autoregulation

Cerebral autoregulation maintains stable perfusion during changes in blood pressure (Cipolla, 2009; Paulson et al., 1990). In healthy cerebrovasculature, CBF is fairly constant over a blood pressure range of 60–150 mmHg (Paulson et al., 1990), whilst CBF varies linearly with blood pressure outside of these ranges, with ischaemia arising in the low pressure limit. MRI can be used to probe cerebral autoregulation mechanisms, such as measuring the effect of a lower‐body negative pressure task on intracranial arterial blood volume (Whittaker, Bright et al., 2019), observing the effect of thigh‐cuff occlusion on oscillations in cerebral blood oxygenation (Whittaker, Steventon et al., 2022) and measuring the effect of natural spontaneous changes in blood pressure on cerebral blood oxygenation (Whittaker, Driver et al., 2019).

### 3.5 Blood–brain barrier

The blood–brain barrier integrity can be measured with MRI by measuring the exchange of an intravascular tracer into brain tissue. This is commonly achieved through intravenous injection of a gadolinium‐based macromolecule (Tofts & Kermode, 1991); however, this method is not sensitive to early stages of blood–brain barrier breakdown (Armitage et al., 2011; Heye et al., 2016; Manning et al., 2021). Recently, several MRI methods for measuring the exchange of water (an endogenous tracer) across the blood–brain barrier have been proposed (Bai et al., 2020; Dickie et al., 2020; Lin et al., 2021, 2018; Ohene et al., 2023; Shao et al., 2019; St. Lawrence et al., 2012; J. Wang et al., 2007; Wells et al., 2013). As a small molecule, exchange of water may indicate breakdown of the blood–brain barrier at an earlier stage than the gadolinium macromolecule. Applications of these methods have found heightened water permeability in ischaemic stroke (Tiwari et al., 2017), sickle cell disease (Lin et al., 2022) and the ageing brain (Ohene et al., 2021), whilst contrarily lower water permeability was observed in obstructive sleep apnoea (Palomares et al., 2015).

### 3.6 Small vessel disease

MRI can detect various aspects of small vessel disease, as reviewed previously (Wardlaw et al., 2013) and summarised as follows. Fluid‐attenuated inversion recovery MRI (FLAIR) is routinely used to detect white matter hyperintensities, which present as diffuse regions of high signal intensity (Melazzini et al., 2021; Wardlaw et al., 2015). Lacunes present with low signal on FLAIR images, but often with a border of high signal. Cerebral microbleeds are visible as areas of low signal on T2*‐weighted MRI due to the magnetic susceptibility of deoxygenated haemoglobin in blood. Small infarcts present as areas of high signal in diffusion‐weighted MRI.

## 4 APPLICATIONS OF MICROVASCULAR MRI MEASURES

The previous section summarised how MRI can measure the brain's microvasculature. This section highlights some applications of these methods to further understanding of the brain in health and disease.

### 4.1 Functional brain networks

By measuring spontaneous activity patterns in the absence of an explicit task (‘resting state’), fMRI has been used to observe a set of functional brain networks which display coordinated activation patterns (S. M. Smith et al., 2009). Measurements of these networks have also been reproduced using magnetoencephalography (Brookes et al., 2011), which measures electrophysiological magnetic field changes and so does not rely on measuring haemodynamic responses to the activation patterns. Recently, a second set of networks have been observed, which have a strong spatial overlap with the functional brain networks, but their temporal characteristics follow arterial CO_2_ (Bright et al., 2020), or breathing depth, which modulates arterial CO_2_ (Chen et al., 2020). As arterial CO_2_ acts as a vasodilator, these networks are considered vascular networks, suggesting an organisation of the cerebrovascular regulation that matches the functional network structure. The origin of this vascular/functional network complementarity is currently unclear. Potential mechanisms could be developmental, with the cerebrovasculature developing to meet the evolving demands of the developing cortex (Black et al., 1990; Quaegebeur et al., 2011; Swain et al., 2003); it could be due to similarities in vascular density across the functional brain network (Vigneau‐Roy et al., 2014); or it could indicate a long‐distance form of vascular signalling, although signalling along smooth muscle cells (Haddock & Hill, 2005) is an unlikely route, as the cerebrovascular structure does not directly link nodes in these networks (Tak et al., 2015), so another signalling method is required (Quaegebeur et al., 2011). Further evidence of these vascular networks was found in a study of CVR in stroke patients (Geranmayeh et al., 2015). CVR was inhibited in stroke lesion regions, compared to healthy tissue and, interestingly, the homologous region in the opposite hemisphere also showed lower CVR compared to healthy tissue. The similar CVR between stroke region and contralateral region is suggestive of a long‐distance vascular network.

### 4.2 Cerebral autoregulation

Our group is interested in how systemic changes in blood pressure affect the cerebrovasculature. A lower body negative pressure task is an orthostatic challenge which reduces pressure in the legs, causing blood to pool in the legs. ASL was used to map arterial blood volume during lower body negative pressure, finding vasoconstriction of large arteries and vasodilatation of smaller vessels (Whittaker, Bright et al., 2019). This provides evidence for the mechanism of cerebral autoregulation, with vascular tone tuned to adapt to changes in systemic blood pressure. Another systemic blood pressure perturbation is a thigh‐cuff release task, which causes a transient reduction in mean arterial pressure. Using BOLD fMRI as a surrogate measurement of CBF, we were able to see a global CBF response to the reduction in mean arterial pressure, providing insight into dynamic cerebral autoregulation (Whittaker, Steventon et al., 2022). Further, there was heterogeneity in the delay of the CBF response, with grey matter leading white matter, but also spatial patterns in the delays in the cortex consistent with the vascular networks observed previously (Bright et al., 2020; Chen et al., 2020). The dynamic cerebral autoregulation relationship between mean arterial pressure and BOLD fMRI can even be observed without a blood pressure challenge, but with spontaneous fluctuations in blood pressure (Whittaker, Driver et al., 2019).

### 4.3 Aerobic exercise

A direct example of where cardiovascular function affects cerebrovascular health is the impact of aerobic exercise on cerebral perfusion. Aerobic exercise is known to have a positive impact on cognition (Erickson et al., 2011; Hillman et al., 2008; Kennedy et al., 2016). Focussing on the hippocampus, which has a key role in memory, hippocampal CBF is increased following 15–20 min of moderate aerobic exercise (Palmer et al., 2023; Steventon, Foster et al., 2020; Steventon, Furby et al., 2020; Vidoni et al., 2022). This acute cerebrovascular response to exercise appears to precede hippocampal angiogenesis (Maass et al., 2015; A. C. Pereira et al., 2007). Hippocampal CBF increases after 1 week (Steventon et al., 2021) and 12 months (Kaufman et al., 2021; Thomas et al., 2020) of moderate aerobic exercise training. Further, there is a positive correlation between hippocampal CBF and aerobic fitness (${\dot{V}}_{O_{2}\max}$) in pre‐adolescent children (Chaddock‐Heyman et al., 2016). However, the data relating perfusion to exercise training are equivocal, with no significant change in CBF after 12 weeks of aerobic exercise training (Chapman et al., 2013; Kaiser et al., 2022; Maass et al., 2015). The ambiguity in results could be due to variability in the underlying fitness of the populations studied, differences in the exercise training or heterogeneity in the ages studied, rather than indicating a 12‐week nadir in the perfusion adaptation to aerobic exercise training. A common theme through these studies is a positive association between memory task performance and hippocampal perfusion (Chapman et al., 2013; Kaufman et al., 2021; Maass et al., 2015; A. C. Pereira et al., 2007; Thomas et al., 2020). Furthermore, the effect of aerobic exercise on hippocampal CBF was augmented in Apolipoprotein E ɛ4 (APOE‐ε4) carriers, compared to non‐carriers (Kaufman et al., 2021; Vidoni et al., 2022). APOE‐ε4 is a major genetic risk factor for Alzheimer's disease, so the exercise‐augmented hippocampal perfusion might be a neuroprotective mechanism in a group vulnerable to a neurodegenerative condition.

Aerobic exercise fitness also affects cerebrovascular reactivity (CVR), which is commonly considered as a surrogate measurement of cerebrovascular health. Cardiorespiratory fitness (CRF) is associated with higher CVR as measured as the change in middle cerebral artery velocity by transcranial Doppler (TCD) ultrasound (E. C. Smith et al., 2021). Contrarily, MRI measurements of BOLD CVR consistently show a lower CVR with higher CRF (DuBose et al., 2022; Gauthier et al., 2015; Intzandt et al., 2020; Thomas et al., 2013). These cross‐sectional studies are supported by a longitudinal exercise intervention study, finding a decrease in BOLD CVR following 12 months of aerobic exercise training (Penukonda et al., 2025). The lower BOLD CVR with increasing CRF has been replicated with CBF CVR (Intzandt et al., 2020), confirming that the BOLD CVR association with CRF is due to CBF CVR, rather than vascular plasticity. TCD measures blood velocity in the middle cerebral artery, whereas BOLD and CBF are sensitive to local changes in tissue perfusion. A possible explanation for the discrepancy between macrovascular (TCD) and microvascular (MRI) CVR was proposed by Thomas et al. (2013), whereby microvascular reactivity to CO_2_ could be reduced by sustained exposure to CO_2_, as occurs during aerobic exercise. In support of this hypothesis, 12 weeks of aerobic exercise training in a group with low baseline fitness resulted in an increase in BOLD CVR (DuBose et al., 2022). A microvascular adaptation of reduced reactivity to CO_2_ would take longer to establish than the short‐term 12 weeks of aerobic exercise, whereas the impact is only seen after 12 months (Penukonda et al., 2025). However, caution should be taken in interpreting the association between CRF and BOLD CVR reported in DuBose et al. (2022), as the BOLD CVR data are presented only as percentage change to the breath hold, not normalising to the actual change in arterial CO_2_. The consequence is that this assumes that the whole population perform and react to the breath hold in the same way, which is not the case (Bright & Murphy, 2013), especially as changes in arterial CO_2_ are likely to be driven by CRF. Interestingly, the effect of aerobic fitness on both macrovascular (TCD) and microvascular (BOLD MRI) CVR only arises in old age (>55 years), with no association observed in young cohorts (Barnes et al., 2013; Intzandt et al., 2020). This suggests that sustained aerobic exercise may partially reverse age‐related changes in cerebrovascular function, which could account for improving cognitive performance following 12 months of aerobic exercise training in participants with amnestic mild cognitive impairment (Penukonda et al., 2025).

### 4.4 Genetic risk factors

Continuing the theme of genetic risk to Alzheimer's disease, MRI measurements of CBF have identified lower grey matter CBF in young, healthy APOE‐ε4 carriers, compared to non‐carriers and established a negative association between CBF and genetic risk of Alzheimer's disease, even once APOE status was removed (Chandler et al., 2019). Furthermore, the negative association between genetic risk and grey matter CBF was also observed in an older cohort (55–85 years), hinting that hypoperfusion throughout the lifetime contributes to risk of Alzheimer's disease (Chandler et al., 2022). A recent study investigating the relationship between Alzheimer's disease genetic risk and white matter hyperintensities found a link between genes over‐expressed in vascular smooth muscle cells and white matter hyperintensity volume (Chandler et al., 2025). This suggests a mechanism related to smooth muscle cells, which regulate vascular tone, and the formation of white matter hyperintensities, which are characteristic of small vessel disease (Wardlaw et al., 2013) and are a risk factor for developing Alzheimer's disease and other dementias.

## 5 MEASURING MACROVASCULAR FUNCTION WITH MRI

Several MRI measurements have been developed to study the large arteries feeding and large veins draining the brain. This section briefly introduces them, before focussing on measurements of arterial stiffness. Table 1 summarises each MRI method and presents their strengths and weaknesses in context of alternative methods.

### 5.1 Cerebral blood flow

Previous sections have focussed on measurements of local perfusion and blood oxygenation; however, global equivalent measurements can be made in the large arteries and veins at the base of the brain. Blood flow through the internal carotid and vertebral arteries can be measured using a technique called phase‐contrast (pc)MRI (Spilt et al., 2002; Stoquart‐ElSankari et al., 2007; Zarrinkoob et al., 2015). The speed of blood can be encoded into the phase of the MRI signal by imposing a spatial gradient in magnetic field along the direction of flow (known as a velocity encoding gradient). The phase of the MRI signal in the blood vessel can be converted to speed based on knowledge of the amplitude, shape and duration of the spatial magnetic field gradient. The speed in cm/s is converted into blood flow in mL/min by multiplying by the cross‐sectional area of the blood vessel. To calculate global CBF, the summed blood flow across internal carotid and vertebral arteries is divided by the total brain volume (Vernooij et al., 2008). There are two common variants of pcMRI, 2D and 4D (Markl et al., 2012). The 2D variant involves acquiring a single 2D slice at a time, positioning the slice to be perpendicular to the vessel(s) of interest. The 4D variant, also known as 4D flow MRI, is a 3D acquisition, so can cover the whole brain. Velocity encoding gradients are applied sequentially in the three cardinal directions, so a blood flow velocity vector can be calculated. The extra spatial dimension and three velocity encoding directions lead 4D flow to have significantly longer acquisition times than 2D pcMRI; however, work on accelerating acquisitions, mostly driven by cardiovascular researchers, has reduced acquisition times to clinically feasible durations (van Schuppen et al., 2024). These pcMRI measurements have been extended to downstream arteries to study how blood flow is distributed across different variants of the circle of Willis (Zarrinkoob et al., 2015). Pushing this method to its limits, blood velocity was measured in perforating arteries in the basal ganglia and centrum semioval (Bouvy et al., 2016), although the conversion to flow was not possible, as reliable estimates of cross‐sectional area were not measurable in these small arteries.

### 5.2 Cerebral oxygen extraction

Blood oxygenation can be calculated based on the magnetic susceptibility (Driver et al., 2014; Fernández‐Seara et al., 2006; Haacke et al., 1997; Jain et al., 2010) or transverse relaxation (Jain et al., 2013; Lu & Ge, 2008; Lu et al., 2012; Oja et al., 1999) in large draining veins. This has been combined with CBF measurements to calculate global cerebral oxygen metabolism (Jain et al., 2010; Xu et al., 2009). Further, these methods can be extended to smaller veins, to restrict oxygen extraction measurements to specific brain regions (Fan et al., 2012; Krishnamurthy et al., 2014). However, these regional measurements are limited by minimal vessel diameters, to avoid the measurements being biased by uncertainty over the vessel size. This can be avoided by calibrating the oxygenation measurements with a hyperoxia challenge (Driver et al., 2014), but these regional measurements have largely been superseded by the microvascular mapping methods described in Section 3.

### 5.3 Arterial stiffness

The large arteries and veins have a role in dampening the cardiac pulse wave as it passes through the brain. Pulsatile flow enters the brain through the feeding arteries and creates pressure waves that propagate through brain tissue (Van Hulst et al., 2024; Wagshul et al., 2011). However, the brain lies within the skull, which acts as a solid container. Blood and cerebrospinal fluid (CSF) are incompressible fluids, so to protect brain tissue from damage from the cardiac pulse pressure waves, the incoming pulse wave is buffered by arterial compliance and transmitted out of the brain through veins and CSF (Bateman et al., 2008). Arteries stiffen with age (Parikh et al., 2016), so the ability of arteries to attenuate the pulse wave is diminished (Chirinos et al., 2019; Lefferts et al., 2020; Tarumi et al., 2014; Zarrinkoob et al., 2016). The strength of the pulse wave entering the brain is regulated by the aortic stiffness (Lefferts et al., 2020), which is generally assessed using carotid–femoral pulse wave velocity (PWV) measurements (Milan et al., 2019; Parikh et al., 2016). The mechanism for the attenuation of the pulse wave within the skull remains to be established. One study observed that the damping of the pulse wave between the common carotid artery and middle cerebral artery (MCA) appears to depend more on the reflections in the pulse wave measured in the common carotid than the forward pulse wave propagation from the aorta (Lefferts et al., 2020). However, this observation lacks a mechanistic underpinning to establish how reflected power could sufficiently dampen the pulse wave (Chirinos et al., 2019).

The increase in carotid–femoral PWV beyond middle‐age (Heffernan et al., 2018; Lefferts et al., 2020; Parikh et al., 2016; Tarumi et al., 2014) is accompanied by increased single‐point common carotid PWV (Lefferts et al., 2020) and internal carotid blood flow velocity pulsatility (Fico et al., 2022; Tarumi et al., 2014), and decreases in carotid pulse wave reflection (Lefferts et al., 2020; Tarumi et al., 2014) and in the damping of the pulse wave between carotid and MCA (Lefferts et al., 2020; Zarrinkoob et al., 2016). There is a negative association between aortic stiffness and memory in older adults, mediated by cerebrovascular resistance through the basilar and internal carotid arteries (Cooper et al., 2016). Further, aortic PWV, and carotid and MCA pulsatility are increased during a cognitive challenge (Stroop task) in older, but not younger adults (Heffernan et al., 2018), suggesting that stiffer arteries are less able to adapt to the stress of increased cognitive load. Arterial stiffness has been linked to small vessel disease (O'Rourke & Safar, 2005; Stone et al., 2015) and white matter hyperintensity volume (Cooper et al., 2016; Tarumi et al., 2014). Whilst these studies are cross‐sectional, and longitudinal studies are needed to establish causality, it is likely that heightened arterial stiffness and reduced damping in cerebral arteries lead to propagation of the pulse wave into microvasculature, damaging the blood–brain barrier and resulting in the formation of white matter hyperintensities. Therefore, measurements of arterial stiffness and propagation of the cardiac pulse wave through intracranial blood vessels are needed to understand the mechanisms linking cardiovascular health to neurodegeneration and to help to measure the efficacy of interventions, such as aerobic exercise.

Arterial compliance (blood volume changes per unit change in pressure) has been measured in the arteries above the circle of Willis (anterior, middle and posterior cerebral arteries) using ASL with a short delay between label and image to capture arterial blood volume, before the label can perfuse into tissue (Warnert et al., 2015). Arterial distensibility (cross‐sectional area of the blood vessel change between diastole and systole) was also measured by the same group, by recording high spatial resolution T_2_‐weighted MRI images synchronised to the cardiac cycle (Warnert, Verbree et al., 2016). Pulsatility wave analysis and pulsatility index can be measured in the large intracranial arteries using pcMRI (Bouillot et al., 2018; Owashi et al., 2023; Rivera‐Rivera et al., 2017) and this has been extended to smaller perforating arteries (Arts et al., 2022; Geurts et al., 2019). However, the key established measurement for arterial stiffness is pulse wave velocity (Alastruey et al., 2023; Marshall et al., 2024; Milan et al., 2019; T. Pereira et al., 2015). MRI has the unique ability to non‐invasively measure the pulsatile flow of arteries deep within the brain, making a local intracranial pulse wave velocity possible. Pulse wave velocity measured across the circle of Willis has been demonstrated using 4D flow MRI (Björnfot et al., 2021), which is a version of pcMRI with magnetic gradients in the three cardinal directions to encode flow velocity, with cardiac gating to resolve the pulse waveform across the average heartbeat. The problem with pcMRI is that the measurement takes several minutes, or hundreds of heartbeats. Natural heart rate variability will smooth out these averaged pulse waveforms, limiting their ability to resolve the fine scale pulse delays of tens of milliseconds between intracranial arteries. In our lab, we have developed an inflow MRI method that is sensitive to flow speed and gives a single image in <15 ms, allowing us to resolve the pulse waveform of a single heartbeat in intracranial arteries (Whittaker, Fasano et al., 2022). This method shows promise for application to calculate the pulse delay as it propagates across the circle of Willis, with potential for measuring beat‐to‐beat pulse wave velocity.

## 6 CONCLUSION: THE IMPACT OF THE CARDIOVASCULAR SYSTEM ON CEREBROVASCULAR HEALTH

The heartbeat provides a constant driving force entering the skull; this is initially buffered by compliant arteries, but as the brain ages, factors like arterial stiffness and blood pressure impair this buffering. The constant insult of the cardiac pulse wave starts to cause tissue damage. It leads to damage to smooth muscle cells and the endothelium. The compromised blood–brain barrier allows macromolecules to enter the brain parenchyma, such as immune cells, which cause multiple sclerosis, or amyloid proteins, leading to dementia. MRI provides a range of tools to measure cerebrovascular health. The next step towards understanding the mechanisms underlying cerebrovascular health and neurodegeneration and to inform intervention decisions will be to study the link between systemic cardiovascular and cerebrovascular physiology.

## AUTHOR CONTRIBUTIONS

Ian Driver: Formal analysis, writing—original draft. Kevin Murphy: Conceptualization, writing—review & editing. Both authors have read and approved the final version of this manuscript and agree to be accountable for all aspects of the work in ensuring that questions related to the accuracy or integrity of any part of the work are appropriately investigated and resolved. All persons designated as authors qualify for authorship, and all those who qualify for authorship are listed. For the purpose of open access, the author has applied a CC BY public copyright licence to any Author Accepted Manuscript version arising from this submission. The authors did not use generative AI in the preparation of this manuscript.

## CONFLICT OF INTEREST

None declared.
